# Symptomatic Dermatographism Misattributed to Cockroach Exposure: A Diagnostic Pitfall

**DOI:** 10.7759/cureus.104837

**Published:** 2026-03-07

**Authors:** Simona Kogan, Sumeet Bhardwaj, Suchana Ghimire, Arushi Tewari, Rishima Tewari

**Affiliations:** 1 Dermatology, Philadelphia College of Osteopathic Medicine (PCOM), Philadelphia, USA; 2 Psychiatry, Kansas City University, Kansas City, USA; 3 Veterinary Medicine, University of Maryland, College Park, College Park, USA; 4 Biology, University of Santa Barbara, Santa Barbara, USA

**Keywords:** allergic reaction, claritin, cockroach exposure, cutaneous hypersensitivity, dermatographia, dermatographism-physical urticaria, diagnostic pitfall, environmental trigger, erythematous rash, pruritus

## Abstract

Allergen exposure can present with a broad clinical spectrum. Individuals exposed to allergens may develop respiratory diseases such as asthma, skin manifestations such as urticaria, and potentially anaphylaxis. For those who reside in urban or unsanitary environments, cockroaches can serve as an unfortunate and persistent exposure to such allergens. Cockroach allergens are classically associated with IgE-mediated respiratory diseases. However, reliance on exposure history alone may lead to diagnostic misattribution when cutaneous symptoms occur without systemic allergic features. We describe a 22-year-old woman from Philadelphia who presented with a 48-hour history of diffuse pruritus and widespread erythematous linear and maculopapular wheals involving the arms, legs, trunk, and face following significant household cockroach exposure. Physical examination demonstrated inducible wheals consistent with symptomatic dermatographism, without evidence of systemic allergic reaction or arthropod envenomation. The temporal association with environmental exposure initially suggested an allergic etiology; however, clinical findings supported a diagnosis of physical urticaria rather than a true allergen-mediated process. Treatment with loratadine 10 mg once daily resulted in symptomatic improvement. This case highlights how dermatographism may be incorrectly attributed to environmental allergens in the setting of coincidental exposure and the importance of careful clinical evaluation to avoid unnecessary allergy-directed testing, particularly in urban settings with high pest prevalence.

## Introduction

Dermatographism is the most common form of physical urticaria, a subgroup of chronic urticaria characterized by the development of wheals or angioedema in response to specific physical stimuli such as pressure or light scratching rather than spontaneously [1]. The pathophysiology involves mechanical stress activating cutaneous mast cells, which release histamine and pro-inflammatory mediators along the area of friction or pressure, producing the characteristic wheal-and-flare response [2,3]. The condition manifests as linear wheals that develop rapidly after mechanical stimulation such as pressure, friction, or light scratching of the skin, typically appearing within 1-3 minutes and lasting 15-30 minutes or longer in symptomatic cases [1,3].

Epidemiologically, dermatographism affects 2%-5% of the general population, though recent international data from the UCARE PREVALENCE-D study of nearly 60,000 participants found an adjusted point prevalence of 3.20% and lifetime prevalence of 5.94%, with higher rates in women and those aged 25-60 years [1,3,4]. Only a minority of affected individuals experience symptoms severe enough to prompt medical attention [1,3].

While histamine is an important mediator, studies suggest that it may not be the sole mediator, particularly in severe cases, and emerging research has identified alterations in gut microbiota and metabolic pathways (including pyruvate, butyric acid, and histamine metabolism) that may contribute to disease pathogenesis [5,6]. The condition is typically idiopathic, though associations with infections (bacterial, fungal, and scabietic), medications (penicillin and famotidine), physiological stress, and atopic conditions have been reported [1,7,8].

Cockroach allergy is well-recognized as a contributor to IgE-mediated respiratory conditions such as asthma and allergic rhinitis, particularly in urban environments with high infestation rates [9]. Proteins derived from *Blattella germanica* are among the most studied cockroach allergens and are known to induce hypersensitivity responses in susceptible individuals, primarily affecting the respiratory tract. While respiratory manifestations are most common, cutaneous symptoms such as pruritus and erythematous eruptions have been reported in heavily infested settings, though these reactions are less well-characterized and may overlap clinically with physical urticarias [10].

In this case, the patient’s widespread pruritic eruption following cockroach exposure highlights how environmental factors may temporally coincide with or nonspecifically exacerbate underlying dermatographism rather than serving as a direct etiologic cause. Although the temporal association between symptom onset and household cockroach exposure initially suggested an allergen-mediated cutaneous reaction, the clinical morphology and reproducible inducibility of the lesions were more consistent with dermatographism rather than a primary allergic dermatitis. Unlike allergic rashes, which are typically antigen-driven and more diffuse or persistent, dermatographism is mechanically induced and transient, reflecting localized mast cell-mediated skin reactivity rather than an environmental allergen response. Reliance on exposure history alone may therefore lead to diagnostic misattribution, particularly in patients residing in cockroach-infested environments. Clinicians practicing in urban settings such as Philadelphia should maintain awareness of this distinction when evaluating rashes of unclear origin, as the careful physical examination and recognition of inducible urticaria can prevent unnecessary allergy testing and the inappropriate attribution of symptoms to environmental allergens [11-13].

## Case presentation

A 22-year-old woman from Philadelphia presented to the Dermatology of Philadelphia / Mohs Surgery Center, LLC with a widespread pruritic eruption that had developed over a 48-hour period. The patient reported a sudden onset of intensely pruritic skin lesions shortly after significant exposure to cockroaches within her home environment. Notably, she had recently moved into a new residence approximately two weeks prior to symptom onset and reported discovering a substantial cockroach infestation shortly after relocating. She described increased stress related to the move and living conditions. She denied any prior history of similar eruptions, chronic urticaria, or dermatographism.

The eruption initially appeared on the bilateral upper and lower extremities and rapidly progressed to involve the trunk and face. The lesions were described as erythematous, raised, and markedly pruritic, leading to frequent scratching. She denied associated systemic symptoms, including fever, chills, arthralgias, respiratory complaints, gastrointestinal symptoms, angioedema, or mucosal involvement.

Her past medical history was unremarkable, with no known chronic illnesses, prior dermatologic conditions, or history of atopic disease. She reported no known drug or environmental allergies and was not taking any prescription or over-the-counter medications prior to presentation. The patient was a nonsmoker, denied alcohol or illicit substance use, and had no recent travel history, sick contacts, or exposure to new personal care products, detergents, or foods. Family history was negative for allergic, autoimmune, or chronic dermatologic conditions.

On physical examination, the patient appeared well-nourished and in no acute distress. Vital signs were within normal limits, including a blood pressure of 120/78 mmHg, heart rate of 72 beats per minute, respiratory rate of 16 breaths per minute, and an oral temperature of 98.6°F (37°C). Cardiopulmonary, abdominal, and neurologic examinations were unremarkable.

**Figure 1:**
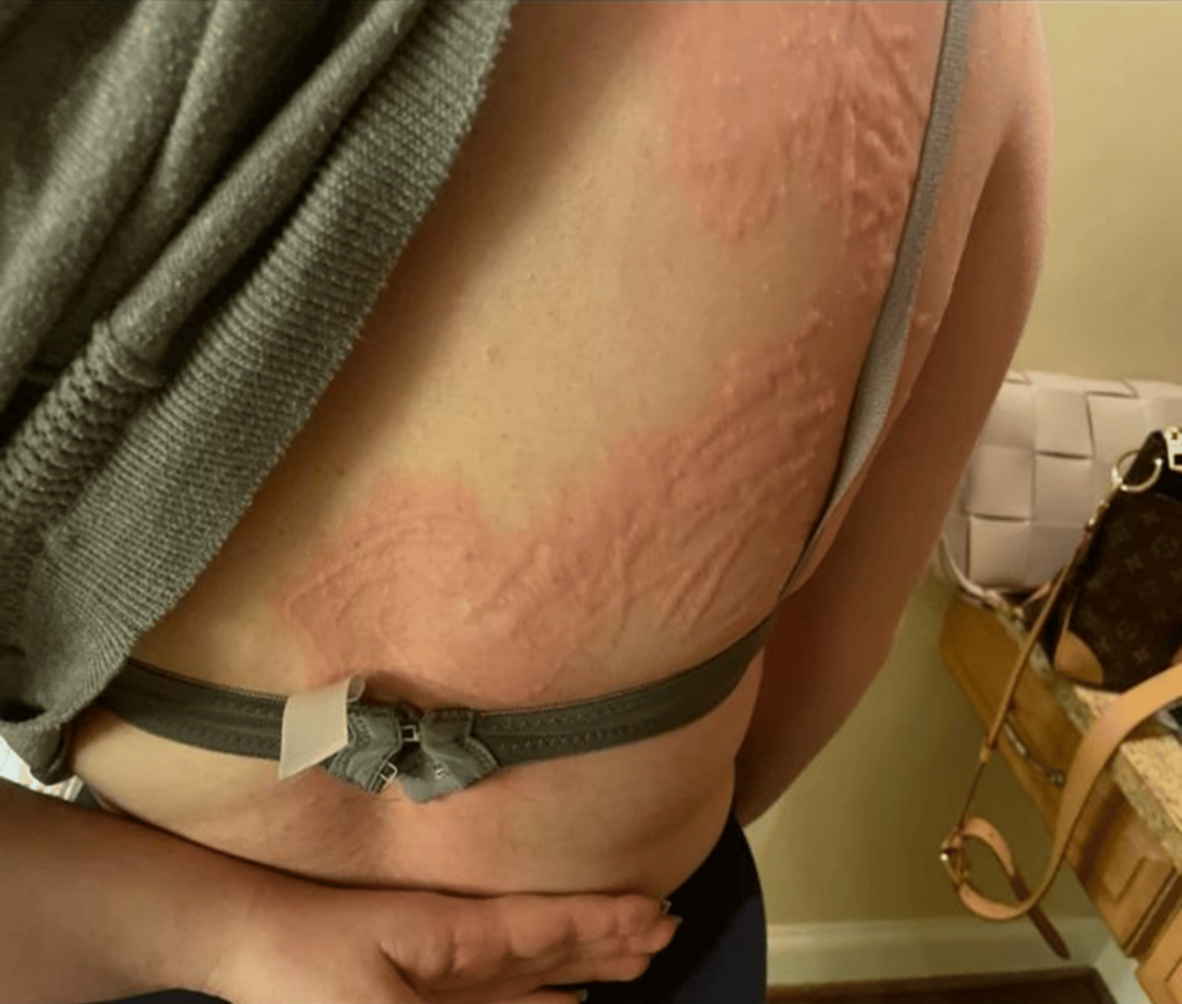
Linear, erythematous wheals along the lateral trunk and upper back consistent with symptomatic dermatographism. Lesions demonstrate a streaked, inducible morphology without central puncta, vesiculation, or features suggestive of arthropod envenomation.

**Figure 2:**
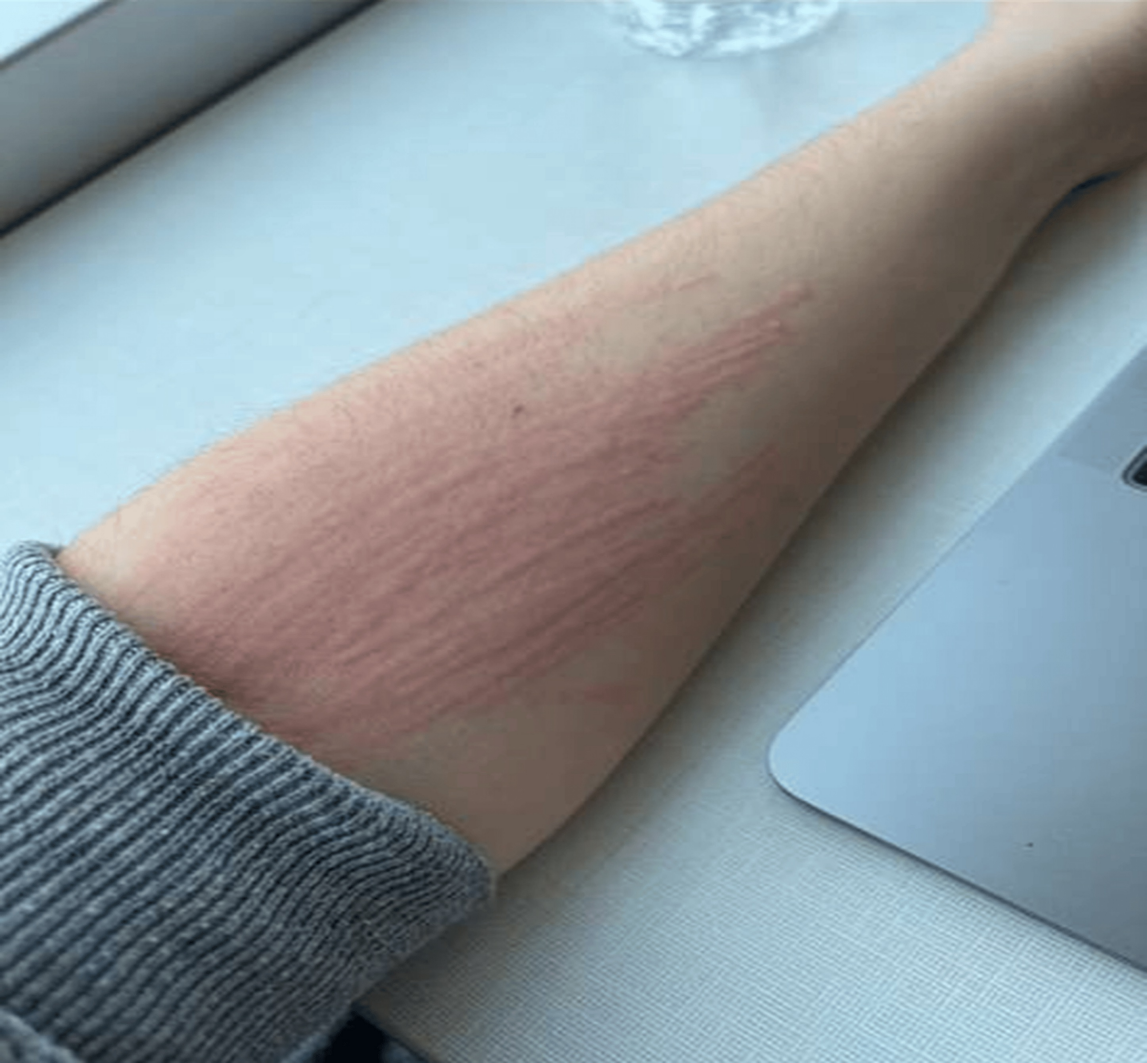
Erythematous linear wheals on the forearm following mechanical stimulation, characteristic of dermatographism (physical urticaria). The distribution and morphology support a non-IgE-mediated process rather than allergic or bite-related dermatitis.

Dermatologic examination revealed widespread erythematous wheals and excoriated plaques distributed over the upper and lower extremities, chest, and back (Figures 1, 2). The lesions appeared raised and linear in configuration in several areas, consistent with mechanical provocation from scratching. Facial involvement was noted; however, there was no periorbital edema, lip swelling, or evidence of angioedema. No vesicles, pustules, central puncta, crusting, or signs suggestive of arthropod envenomation were observed. Mucosal surfaces were spared, and there was no evidence of secondary infection.

Given the acute onset of symptoms shortly after relocating to a new residence with a significant cockroach infestation, an allergic etiology was initially considered. The patient reported that her previous residence had no known pest issues and that the infestation in the new home was both unexpected and substantial, representing a new environmental exposure. However, the morphology of the lesions and their reproducible, inducible nature on physical examination were more consistent with physical urticaria (dermatographism) rather than a primary allergen-mediated dermatitis.

She was treated with loratadine 10 mg once daily and counseled on environmental remediation measures, including the eradication of the infestation. At two-week follow-up, she reported marked improvement in pruritus with near-complete resolution of wheals. No recurrence or progression of symptoms was observed, further supporting the diagnosis of symptomatic dermatographism.

Although formal dermographic provocation was not performed, multiple wheals were observed along areas of recent scratching, demonstrating a linear wheal-and-flare pattern clinically consistent with symptomatic dermatographism. Individual lesions were transient by history and morphology and were reported by the patient to resolve within several hours without residual skin change. Given the classic presentation and absence of systemic features suggestive of alternative etiologies, laboratory evaluation was deferred. Limitations include reliance on clinical assessment without formal provocation testing or laboratory confirmation.

## Discussion

Cockroach allergy is a major environmental concern, particularly in urban areas with high infestation rates, and is most commonly associated with IgE-mediated respiratory conditions such as asthma and allergic rhinitis [14]. In contrast, cutaneous manifestations related to cockroach exposure are less frequently discussed in the literature and may be prone to diagnostic misattribution. In this case, the patient reported diffuse pruritus following household cockroach exposure, initially raising concern for an allergen-mediated skin reaction. However, the wheals observed on examination were linear and reproducible with mechanical stimulation, consistent with symptomatic dermatographism rather than spontaneous generalized urticaria or primary allergic dermatitis. While dermatographism is most often idiopathic, the environmental exposure was considered temporally associated rather than causative.

Cockroach-derived proteins from exoskeletons, saliva, and fecal matter are recognized allergens and may act as nonspecific environmental stressors capable of provoking pruritus or lowering the threshold for mast cell activation in susceptible individuals [12,15]. While *Blattella germanica* is a well-established source of sensitization in atopic individuals, the absence of prior allergic reactions in this patient and the lack of systemic features argue against a true IgE-mediated cutaneous allergy [16]. Environmental infestation may nevertheless contribute to symptom amplification through mechanical irritation, scratching, or neuroimmune pathways, thereby unmasking previously subclinical dermatographism [17].

Although cockroach bites and transmigrant contact have been described as potential causes of localized skin reactions, including urticaria and dermatitis, the existing literature remains limited and largely anecdotal [11]. Reported cutaneous findings such as erythematous patches, papules, or plaques may overlap clinically with physical urticaria, further complicating diagnosis. In this context, reliance on exposure history alone may bias clinicians toward an allergic or envenomation-based explanation, despite clinical features that are more consistent with inducible urticaria.

The diagnosis of symptomatic dermatographism was made clinically based on reproducible wheal formation with mechanical stimulation. Allergen-specific IgE testing was not performed, as there were no systemic features suggestive of allergic disease. Dermatographism is typically idiopathic, and the environmental exposure in this case was considered temporally associated rather than causative. Testing for cockroach-specific IgE is not routinely indicated in isolated cutaneous presentations and is generally reserved for recurrent, severe, or systemic allergic disease [13,18]. Symptomatic management with loratadine 10 mg once daily for two weeks resulted in improvement, consistent with standard treatment for physical urticaria [12]. Environmental control measures were discussed as part of general symptom mitigation rather than as the treatment of a proven allergic etiology.

This case underscores the importance of distinguishing dermatographism from allergen-mediated skin disease in patients presenting with pruritic eruptions following environmental exposure. In urban settings such as Philadelphia, where cockroach infestation is common, the heightened awareness of this diagnostic pitfall may help clinicians avoid unnecessary allergy testing and the inappropriate attribution of symptoms to environmental allergens. The greater recognition of physical urticaria in dermatologic and primary care practice may lead to more accurate diagnosis and more effective, targeted management [11].

Disclaimer

If symptoms persist despite the empiric modification of a potential environmental exposure, the patient should be reassessed and undergo a more comprehensive evaluation. In such cases, the possibility of idiopathic dermatographism should be considered, particularly when symptoms continue despite allergen avoidance, and management may require targeted therapy such as antihistamines rather than environmental modification alone.

## Conclusions

This case underscores the importance of including dermatographism in the differential diagnosis of acute pruritic eruptions occurring after environmental exposure. Although such presentations may raise concern for allergen-mediated reactions, particularly in settings where environmental allergens are prevalent, the clinical findings in this case were more consistent with physical urticaria. The recognition of characteristic features and response to non-sedating antihistamines, such as loratadine, supported the diagnosis and allowed for effective symptom control. While environmental mitigation strategies may contribute to overall health, careful clinical evaluation is critical to avoid diagnostic misattribution and unnecessary allergy-directed investigations.
